# Segmentor3IsBack: an R package for the fast and exact segmentation of Seq-data

**DOI:** 10.1186/1748-7188-9-6

**Published:** 2014-03-10

**Authors:** Alice Cleynen, Michel Koskas, Emilie Lebarbier, Guillem Rigaill, Stéphane Robin

**Affiliations:** 1 AgroParisTech, UMR 518, 16 rue Claude Bernard, 75231 Paris Cedex 05, France; 2 INRA, UMR 518, 16 rue Claude Bernard, 75231 Paris Cedex 05, France; 3, Unité de Recherche en Génomique Végétale (URGV) INRA-CNRS-Université d’Evry Val d’Essonne, 2 Rue Gaston Crémieux, 91057 Evry Cedex, France

**Keywords:** Segmentation algorithm, Exact algorithm, Fast algorithm, RNA-Seq data, Genome annotation, Count data, Data compression

## Abstract

**Background:**

Change point problems arise in many genomic analyses such as the detection of copy number variations or the detection of transcribed regions. The expanding Next Generation Sequencing technologies now allow to locate change points at the nucleotide resolution.

**Results:**

Because of its complexity which is almost linear in the sequence length when the maximal number of segments is constant, and as its performance had been acknowledged for microarrays, we propose to use the Pruned Dynamic Programming algorithm for Seq-experiment outputs. This requires the adaptation of the algorithm to the negative binomial distribution with which we model the data. We show that if the dispersion in the signal is known, the PDP algorithm can be used, and we provide an estimator for this dispersion. We describe a compression framework which reduces the time complexity without modifying the accuracy of the segmentation. We propose to estimate the number of segments via a penalized likelihood criterion. We illustrate the performance of the proposed methodology on RNA-Seq data.

**Conclusions:**

We illustrate the results of our approach on a real dataset and show its good performance. Our algorithm is available as an *R* package on the CRAN repository.

## Background

Change-point detection methods have long been used in the analysis of genetic data as an efficient tool in the study of DNA sequences for various purposes. For instance, segmentation methods have been developed for categorical variables with the aim of identifying patterns for gene predictions [1,2], while SNPs have been detected using sequence segmentation [3]. In the last two decades, with the large spread of micro-arrays, change-point methods have been widely used for the analysis of DNA copy number variations and the identification of amplification or deletion of genomic regions in pathologies such as cancer [4-8].

The recent development of Next-Generation Sequencing technologies gives rise to new applications along with new difficulties: (*a*) the increased size of profiles (up to 10^8^ data-points when micro-array signals were closer to 10^5^), and (*b*) the discrete nature of the output (number of reads starting at each position of the genome). Yet applying segmentation methods to DNA-Seq data with their greater resolution should lead to the analysis of copy-number variation with a much improved precision compared to CGH arrays. Moreover, in the case of poly-(A) RNA-Seq data on lower organisms, since coding regions of the genome are well separated from non-coding regions with lower activity, segmentation methods should allow the identification of transcribed genes as well as address the issue of new transcript discovery. Our objective is therefore to develop a segmentation method to tackle both (*a*) and (*b*) with some specific requirements: the amount of reads falling within a segment should be representative of the biological information associated (relative copy-number of the region, relative level of expression of the gene), and comparison to neighboring regions should be sufficient to label the segment (for instance normal or deleted region of the chromosome in DNA-Seq data, exon or non-transcribed region in RNA-Seq), therefore no comparison profile should be needed. This also suppresses the need for normalization, and consequently we wish to analyze the raw count-profile.

Up to now, most methods addressing the analysis of these datasets require some normalization process to allow the use of algorithms which rely on Gaussian-distributed data or which were previously developed for micro-arrays [9-12]. Indeed, methods adapted to count datasets are not numerous and are highly focused on the Poisson distribution. Alteration of genomic sequences can be detected based on the comparison of Poisson processes associated with the read counts of a case and a control sample [13], but this cannot be applied to the detection of transcribed regions in a single condition.

Still, a likelihood ratio statistic was proposed for the localization of a shift in the intensity of a Poisson process [14], and a test statistic was proposed for the existence of a change-point in the Poisson autoregression of order 1 [15].

These last two methods do not require a comparison profile but they only allow for the detection of a single change-point and have too high a time-complexity to be applied to RNA-Seq profiles. Binary Segmentation, a fast heuristic [6], and Pruned Exact Linear Time (PELT) [16], an exact algorithm for optimal segmentation with respect to the likelihood, are both implemented for the Poisson distribution in the **changepoint** package. Even though both are extremely fast, do not require a comparison profile, and analyze count-data, the Poisson distribution is not adapted to our kind of datasets.

A recent study [17] has compared 13 segmentation methods for the analysis of chromosomal copy number profiles and has shown the excellent performance of the Pruned Dynamic Programming (PDP) algorithm [18] proposed in its initial implementation for the analysis of Gaussian data in the *R* package **cghseg**. We propose to use this algorithm, which we have implemented for the Poisson and negative binomial distributions.

In the next section we recall the general segmentation framework and the definition and requirements of the PDP algorithm. Our contributions are given in the third section where we define the negative binomial model and show that it satisfies the PDP algorithm requirements. We also provide a theoretical result for the possibility to compress the data, and finally we give a model selection criterion with theoretical guarantees, which makes the whole approach complete. We conclude with a simulation study, which illustrates the performance of the proposed method.

## Segmentation model and algorithm

### General segmentation model

The general segmentation problem consists in partitioning a signal of *n* data-points {*y*_*t*_}_*t*∈[ [1,*n*] ]_ into a given number *K* of pieces or segments. The model can be written as follows: the observed data {*y*_*t*_}_*t*=1,…,*n*_ are supposed to be a realization of an independent random process *Y*={*Y*_*t*_}_*t*=1,…,*n*_. This process is drawn from a probability distribution  which depends on a set of parameters among which one parameter *θ* is assumed to be affected by *K*-1 abrupt changes, called change-points, such that

$$Y_{t}\sim G\left(\theta_{r},,,\phi \right)\;\text{if}\;t\;\in \;r\;\text{and}\;r\in m$$

where *m* is a partition of [ [1,*n*] ] into segments *r*, *θ*_*r*_ stands for the parameter of segment *r* and *ϕ* is constant. The objective is to estimate the change-points or the positions of the segments and the parameters *θ*_*r*_ both resulting from the segmentation. More precisely, we define ${ℳ}_{k,t}$ the set of all possible partitions in *k*>0 regions of the sequence up to point *t*. We recall that the number of possible partitions is

cardℳk,t=t-1k-1.

We aim at choosing the partition in ${ℳ}_{K,n}$ of minimal loss *γ*, where the loss is usually taken as the negative log-likelihood of the model. We define the point-additive loss of a segment with given parameter *θ* as $c\left(r,,,\theta \right)=\underset{i\;\in \;r}{\sum }\gamma \left(y_{i},,,\theta \right)$, therefore its optimal cost is *c*(*r*)= min*θ*{*c*(*r*,*θ*)}. This allows us to define the cost of a segmentation *m* as $\underset{r\;\in \;m}{\sum }c(r)$ and our goal is to recover the optimal segmentation *M*_*K*,*n*_ and its cost *C*_*K*,*n*_ which are particular cases of the generic optimal segmentation of the signal up to point *t* in *k* segments and its cost, defined as:

$$\begin{array}{lc} \;M_{k,t}={\arg\min}_{\left\{m,\;,\in ,\;,{ℳ}_{k,t}\right\}}\left\{\underset{r\;\in \;m}{\sum }c\left(r\right)\right\}\\ \text{and}\;C_{k,t}={\text{min}}_{\left\{m,\;,\in ,\;,{ℳ}_{k,t}\right\}}\left\{\underset{r\;\in \;m}{\sum }c\;\left(r\right)\right\}. & \end{array}$$

### Quick overview of the PDP algorithm

Like the original DP algorithm, the pruned DP algorithm is an iterative algorithm based on the minimization of a cost function *C*_*k*,*t*_ which is traditionally decomposed as:

(1) $$\begin{array}{l} C_{k,t}=\underset{\left\{k,-,1,<,\tau ,<,t\right\}}{\min}\left\{C_{k-1,\tau }+\underset{\theta }{\min}\left[c\left(\left[\;,\tau ,+,1,,,t\right],\;,,,\theta \right)\right]\right\} \end{array}$$

where *θ* is the parameter of the cost of the last segment, constraints on its possible values being directly related to the support of the loss function *γ* (for instance *θ* takes its value in  in the case of the Gaussian loss, but in [ 0,1] in the case of the binomial loss). In what follows we will denote by *I*_*s*_ the set of possible values for parameter *θ*.

The specificity of the PDP algorithm is that it relies on the comparison of candidates for the last change-point position *τ* through the permutation of the minimizations in (1) and the introduction of the functions:

$$\begin{array}{l} H_{k,t}\left(\theta \right)=\underset{k-1<\tau \leq t}{\min}\left\{C_{k-1,\tau }+\;c\left(\left[\;,\tau ,+,1,,,t\right],\;,,,\theta \right)\right\}, \end{array}$$

which are the cost of the best partition in *k* regions up to *t*, the parameter of the last segment being *θ*. *C*_*k*,*t*_ is then obtained as min*θ*{*H*_*k*,*t*_(*θ*)}.

Then at each iteration *k*, the PDP algorithm works on a list of last change-point candidates: ListCandidate_*k*_. For each of these *τ*s and for each value of *t*, it updates the set of *θ*s, denoted $S_{k,t}^{\tau }$ for which this candidate is optimal. If this set is empty, the candidate is discarded, resulting in the pruning and lower complexity of the algorithm.

The foundations of the algorithm can be written as follows. 

• Defining $H_{k,t}^{\tau }\left(\theta \right)=C_{k-1,\tau }+\sum_{j=\tau +1}^{t}\gamma \;\left(y_{j},,,\theta \right)$ the optimal cost if the last change is *τ* and last parameter is *θ*, then 

(i) $H_{k,t+1}^{\tau }\left(\theta \right)$ is obtained from $H_{k,t}^{\tau }\left(\theta \right)$ using:

$$\begin{array}{c} \;H_{k,t+1}^{\tau }\left(\theta \right)=H_{k,t}^{\tau }\left(\theta \right)+\left(y_{t+1},,,\theta \right); \end{array}$$

• Defining $I_{k,t}^{\tau }=\left\{\theta \;|\;H_{k,t}^{\tau }\left(\theta \right)\;\leq \;H_{k,t}^{t}\left(\theta \right)\;\right\}=\left\{\theta \;|\;H_{k,t}^{\tau }\left(\theta \right)\;\leq \;C_{k-1,t}\;\right\}$ the set of *θ* such that *τ* is better than *t* in terms of cost, with *τ*<*t*, then 

(ii) if all $\sum_{j=\tau +1}^{t}\gamma \;(y_{j},\theta )$ are unimodal in *θ* then $I_{k,t}^{\tau }$ are intervals. Indeed, since by definition $H_{k,t}^{t}(\theta )=C_{k-1,t}$ and the cost function does not depend on *θ*, $I_{k,t}^{\tau }$ is the set of values for which a unimodal function is smaller than a constant.

• Finally, we introduce $S_{k,t}^{\tau }=\left\{\theta \;|\;H_{k,t}^{\tau }\left(\theta \right)\;\leq \;H_{k,t}\left\{\theta \right\}\right\}$ the set of *θ* such that *τ* is optimal. Then since $H_{k,t}(\theta )=\underset{\tau \leq t}{\min}\left\{H_{k,t}^{\tau }(\theta )\right\}$, $S_{k,t}^{\tau }$ can be written as $\left\{\theta \;|\;H_{k,t}^{\tau }(\theta )\;=\;H_{k,t}(\theta )\;\right\}$ and we obtain that 

(iii) $S_{k,t+1}^{\tau }$ can be updated using: 

⋆ $S_{k,t+1}^{\tau }=S_{k,t}^{\tau }\;\cap I_{k,t+1}^{\tau }$

⋆ $S_{k,t}^{t}=I_{s}\setminus (\cup_{\tau \in {\text{ListCandidate}}_{k}}I_{k,t}^{\tau })$

The first assertion follows from the fact that $S_{k,t+1}^{\tau }=\left\{\theta |H_{k,t+1}^{\tau }\leq \underset{k\leq \tau \leq t+1}{\min}H_{k,t+1}^{\tau }\right\}=\left\{\theta |H_{k,t+1}^{\tau }\leq \min\left(\overset{t+1}{\underset{k,t+1}{H}},\underset{k\leq \tau \leq t}{\min}H_{k,t+1}^{\tau }\right)\right\},$ the first term in the minimum giving $I_{k,t+1}^{\tau }$ and the second one giving $S_{k,t}^{\tau }$. The second assertion trivially follows from the fact that candidate *t* is optimal on the set of values where no other candidate was optimal.

• 

(iv) once it has been determined that $S_{k,t}^{\tau }$ is empty, it easily follows from the update equation (*iii*) that the region-border *τ* can be discarded from the list of candidates *ListCandidate*_*k*_:

$$\begin{array}{lcrr} & S_{k,t}^{\tau }=\emptyset & \Rightarrow \;\forall \;t^{\prime }\geq t\;S_{k,t^{\prime }}^{\tau }=\mathrm{\emptyset .} & \end{array}$$

#### 

##### Requirements of the pruned dynamic programming algorithm

###### Proposition 0.1

Properties (*i*) to (*iv*) are satisfied as soon as the following conditions on the loss *c*(*r*,*θ*) are met: 

(a) It is point additive,

(b) It is convex with respect to its parameter *θ*,

(c) It can be stored and updated efficiently.

The proof of those claims can be found in [18]. A pseudo-code of the PDP algorithm is given in the appendix.

It is possible to include an additional penalty term, denoted *g* as in the pseudo-code, in the loss function. To preserve the point-additivity requirement of the loss, this penalty can only depend on the value of the segment-parameter *θ* and not on any other characteristics, such as segment length. This is then equivalent to minimizing *C*_*k*,*t*_= min{*k*-1<*τ*<*t*}{*C*_*k*-1,*τ*_+ min*θ*{*c*([ *τ*+1,*t*],*θ*)+*g*(*θ*)}} and can be achieved by adding the penalty value *g*(*θ*) in the initialization of $H_{k,t}^{\tau }(\theta )$. For example, in the case of RNA-seq data one could add a lasso (*λ*|*θ*|) or ridge penalty (*λθ*^2^) to encode that *a priori* the coverage in most regions should be close to 0. Our C++ implementation of the PDP algorithm includes the possibility of adding such a penalty term; however we do not provide an R interface to this functionality in our R package. One of the reasons for this choice is that choosing an appropriate value for *λ* is not a simple problem.

## Contribution

### Pruned dynamic programming algorithm for count data

We now show that the PDP algorithm can be applied to the segmentation of RNA-Seq data using a negative binomial model and we propose a criterion for the choice of *K*. Though not discussed here, our results also hold for the Poisson segmentation model.

#### 

##### Negative binomial model

We consider that in each segment *r* all *y*_*t*_ are the realization of random variables *Y*_*t*_ which are independent and follow the same negative binomial distribution. Assuming the dispersion parameter *ϕ* to be known, we will use the natural parametrization from the exponential family (also classically used in *R*) so that parameter *θ*_*r*_ will be the probability of success. In this framework, *θ*_*r*_ is specific to segment *r* whereas *ϕ* is common to all segments.

We have *E*(*Y*_*t*_)=*ϕ*(1-*θ*)/*θ* and *Var*(*Y*_*t*_)=*ϕ*(1-*θ*)/*θ*^2^. We choose the loss as the negative log-likelihood associated with data-point *t* belonging to segment *r*: -*ϕ* log(*θ*_*r*_)-*y*_*t*_ log(1-*θ*_*r*_)+*A*(*ϕ*,*y*_*t*_), or more simply *γ*(*y*_*t*_,*θ*_*r*_)=-*ϕ* log(*θ*_*r*_)-*y*_*t*_ log(1-*θ*_*r*_) since *A* is a function that does not depend on *θ*_*r*_.

#### 

##### Validity of the pruned dynamic programming algorithm for the negative binomial model

###### Proposition 0.2

Assuming parameter *ϕ* to be known, the negative binomial model satisfies (a), (b) and (c):

(a) As we assume that *Y*_*t*_ are independent, we indeed have that the loss is point additive: $c(r,\theta )=\underset{t\;\in \;r}{\sum }\gamma (y_{t},\theta ).$

(b) As *γ*(*y*_*t*_,*θ*)=-*ϕ* log(*θ*)-*y*_*t*_ log(1-*θ*) is convex with respect to *θ*, *c*(*r*,*θ*) is also convex as the sum of convex functions.

(c) Finally, we have $c(r,\theta )=-n_{r}\phi \log(\theta )+\underset{t\;\in \;r}{\sum }y_{t}\log(1-\theta )$ (where *n*_*r*_ is the length of segment *r*). This function can be stored and updated using only two doubles: one for -*n*_*r*_*ϕ*, say *d*_1_, and the other for $\underset{t\;\in \;r}{\sum }y_{t}$, say *d*_2_. Then at step *t*+1 as the new datapoint *y*_*t*+1_ is considered, these doubles are simply updated as *d*_1_←*d*_1_+*ϕ* and *d*_2_←*d*_2_+*y*_*t*+1_.

#### 

##### Estimation of the overdispersion parameter

We propose to estimate *ϕ* using a modified version of Johnson *et. al*’s estimator [19]: compute the moment estimator of *ϕ* on each sliding window of size *h* using the formula $\phi =E{\left(Y\right)}^{2}/(\text{Var}(Y)-E(Y))$ and keep the median $\hat{\phi }$.

#### 

##### Taking into account a positional bias

It is possible that the assumption that the counts share the same distribution in a segment might not be verified. For instance in the case of RNA-Seq data the number of reads can be affected by the location in the transcribed region or by the GC-content of the fragment. The pruned dynamic programming algorithm only requires a vector of integers as input, it is therefore possible to apply any kind of normalization process that preserves the count-specificity of the data prior to segmentation. For instance, a method such as that which has resulted in the publication of the data used in the illustration [20] can be applied. A comparison of the main normalization methods can for example be found in Bullard *et. al.*’s paper [21].

### C++ implementation of the PDP algorithm

We implemented the PDP algorithm in C++ having in mind the possibility of adding new loss functions in potential future applications. The difficulties we had to face were the versatility of the program to be designed and the design of the objects it could work on. Indeed, the use of full templates implied that we used stable sets of objects for the operations that were to be performed.

Namely: 

• The sets were to be chosen in a *tribe*. This means that they all belong to a set S of sets such that any set s∈S can be conveniently handled and stored in the computer. A set of sets S is said to be *acceptable* if it satisfies the following: 

1. If *s* belongs to S, R∖s∈S

2. If $s_{1},s_{2}\in S,\;\;s_{1}\cap s_{2}\in S$

3. If $s_{1},s_{2}\in S,\;\;s_{1}\cup s_{2}\in S$

• For instance, the set S of intervals is a tribe since the complementary, the union and the intersection of intervals form a union of intervals. This property ensures that the sets $I_{k,t}^{\tau }$ and $S_{k,t}^{\tau }$ can be updated and stored efficiently (only two doubles are required to store an interval) to take full advantage of the pruning process.

• The cost functions were chosen in a set F such that 

1. Each function may be conveniently handled and stored by the software.

For instance, for the Gaussian loss it suffices to store the three coefficients of a second order polynomial.

2. For any f∈F and any constant *c*, *f*(*x*)≤*c* can be easily solved and the set of solutions belongs to an acceptable set of sets

3. For any f,g∈F,f+g∈F.

These two points ensure that the cost (and penalty) functions can be easily updated and compared so that the sets $I_{k,t}^{\tau }$ of each candidate *τ* can be updated and candidates eventually discarded.

Thus we defined two collections for the sets of sets S, intervals and parallelepipeds, and implemented the loss functions corresponding to negative binomial, Poisson or normal distributions. The program is thus designed in a way that any user can add his own cost function or acceptable set of probability function and use it without rewriting a line in the code.

### Compression of the signal

In the case of count data, and in particular in the analysis of RNA-Seq data, it is very likely that we observe plateaux, that is regions between two arbitrary positions *t*_1_ and *t*_2_ (>*t*_1_) where the signal is constant:

$$\forall t,\;t_{1}\leq t\leq t_{2},\;y_{t}=y_{t_{1}}=y_{t_{2}}.$$

Then we have the following proposition, the proof of which is given in the appendix.

#### Proposition 0.3

There exists a segmentation *m* in *K* or fewer segments without any change-point in the plateaux such that the optimal cost of *m* is equal to *C*_*K*,*n*_.

This proposition proves the arguably intuitive idea that having a change-point between *t*_1_ and *t*_2_ is never beneficial in terms of cost. When searching for the best segmentation of the data, it is therefore unnecessary to look for change-points in plateaux. In other words a plateau starting at position *t*_1_ and ending at position *t*_2_ can be considered as a unique data point with value $y_{t_{1}}$ and weight *t*_2_-*t*_1_+1. At worst the size of the compressed signal is equal to the minimum between two times the number of reads and the length of the chromosome arm. Thus, if the number of reads is very large, the two-step algorithm (compression and pruned dynamic programming) does not change the worst case complexity. However, in most cases the number of reads is much smaller than the size of the considered chromosome. Thus compression is efficient and allows for a significant reduction in the overall run-time. Furthermore, in the case of RNA-Seq data we do not expect reads to be evenly scattered. On the contrary they are concentrated in transcribed regions and between those regions we expect large plateaux of 0 allowing for an efficient compression (for instance only 2% of the human chromosome contains coding regions).

### Model selection

The last issue concerns the estimate of the number of segments *K*. This model selection issue can be solved using a penalized log-likelihood criterion for which the choice of a good penalty function is crucial. This kind of procedure typically requires computation of the optimal segmentations in all *k*=1,…,*K*_max_ segments where *K*_max_ is generally chosen smaller than *n*. The most popular criteria (AIC [22] and BIC [23]) failed in the segmentation context due to the discrete nature of the change-points. Indeed, additionally to being an asymptotic criterion in a framework where the collection of possible models grows polynomially with *n*, the BIC criterion uses a Laplace approximation requiring differentiability conditions of the likelihood function which are not satisfied by the segmentation model [24]. From a non-asymptotic point of view and for the negative binomial model, the following criterion was proposed [25]: denoting ${\hat{m}}_{K}$ the optimal segmentation of the data in *K* segments,

(2) $$\;\begin{array}{l} \hat{K}\;=\;\underset{K\in 1:K_{\max}}{\arg\;\min}\;\;\left\{\;\underset{r\in {\hat{m}}_{K}}{\sum }\;\underset{t\in r}{\sum }\;\left[-\phi \text{log}\frac{\phi }{\phi +{\bar{y}}_{r}}-y_{t}\log\;\left(\;1-\frac{\phi }{\phi \;+\;{\bar{y}}_{r}}\;\right)\;\right]\;\right)\\ \left(\;+\;\mathrm{\beta K}\;{\left(1+4\sqrt{1.1+\log\left(\frac{n}{K}\right)}\right)}^{2}\right\}, \end{array}$$

where ${\bar{y}}_{r}=\frac{\underset{t\in r}{\sum }y_{t}}{{\hat{n}}_{r}}$ and ${\hat{n}}_{r}$ is the size of segment *r*. The first term corresponds to the cost of the optimal segmentation while the second is a penalty term which depends on the dimension *K* and on a constant *β* that has to be tuned according to the data (see the next section). With this choice of penalty, a so-called oracle penalty, the resulting estimator satisfies an oracle-type inequality. A more complete performance study is done in [25] and showed that the proposed criterion outperforms the existing ones.

## Implementation

The Pruned Dynamic Programming algorithm is available in the function Segmentor of the *R* package **Segmentor3IsBack**. Version 1.7 of this package contains the compression process which is performed by default in the case of count data. The user can choose the distribution with the slot model (1 for Poisson, 2 for Gaussian homoscedastic, 3 for negative binomial and 4 for segmentation of the variance). It returns an S4 object of class Segmentor which can later be processed for other purposes. The function SelectModel provides four criteria for choosing the optimal number of segments: AIC [22], BIC [23], the modified BIC [24] (available for Gaussian and Poisson distribution) and oracle penalties (available for the Gaussian distribution [26] and for the Poisson and negative binomial [25] as described previously). This latter kind of penalty requires tuning a constant according to the data, which is done using the slope heuristic [27].

**Figure 1:**
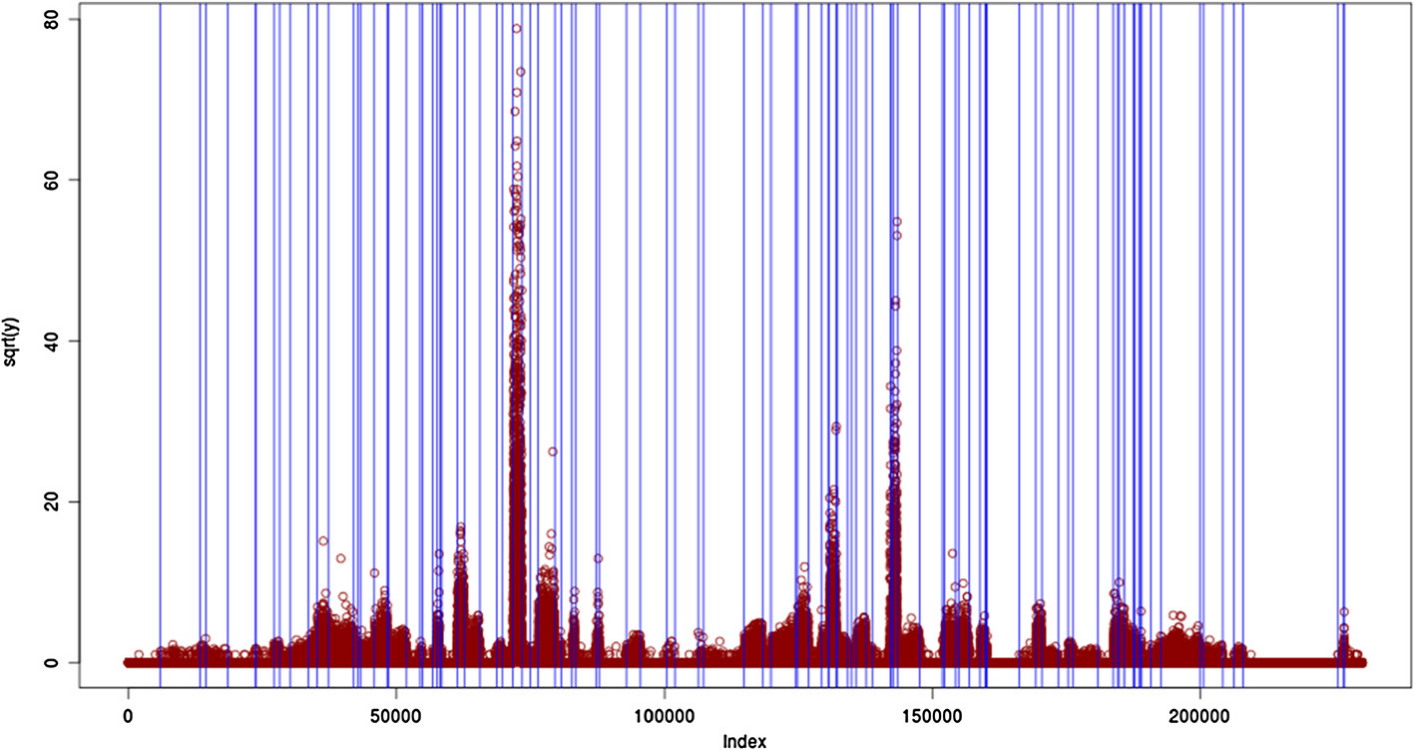
**Segmentation of the yeast chromosome 1 using the negative binomial loss.** The model selection procedure chooses *K*=125 segments, most of which correspond to the official annotation, with segments corresponding to transcribed regions surrounding official genes.

Figure 1 (which is detailed in the Results and discussion Section) was obtained with the following 4 lines of code (assuming the data was contained in vector x):

Seg<-Segmentor(x,model=3,Kmax=200)

Kchoose<-SelectModel(Seg, penalty=~oracle~)

plot(sqrt(x),col=’dark red’)

abline(v=getBreaks(Seg)[Kchoose, 1:Kchoose],col=’blue’)

**Figure 2:**
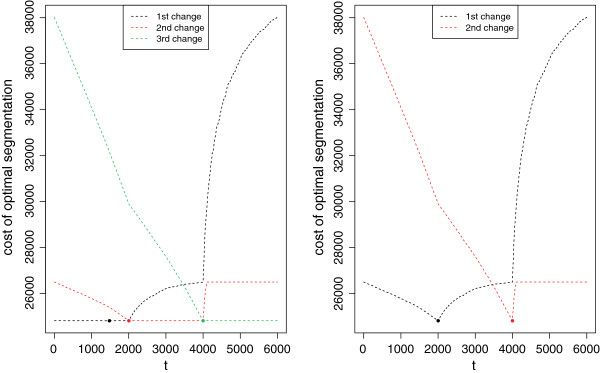
**Cost of optimal segmentation in 4 and 3 segments.** Cost of optimal segmentation depending on the location of the *j*^*th*^ change-point when the number of segments is 4 (Left) and 3 (Right) and the signal was simulated with 3 segments. Illustration of the output of function BestSegmentation.

The function BestSegmentation allows us, for a given *K*, to find the optimal segmentation with a change-point at location *t* (slot $bestSeg). It also provides, through the slot $bestCost, the cost of the optimal segmentation with *t* for *j*^*th*^ change-point. Figure 2(Left) illustrates this result for the optimal segmentations in 4 segments of a signal simulated with only 3 segments. We can see for instance that any choice of first change-point location between 1 and 2000 yields almost the same cost (the minimum is obtained for *t*=1481), and thus the optimal segmentation is not clearly better than the next best segmentations. On the contrary, the same function with 3 segments shows that the optimal segmentation outperforms all other segmentations in 3 segments.

## Results and discussion

### Performance study

We designed a simulation study on the negative binomial distribution to assess the performance of the PDP algorithm in terms of computational efficiency without using the compression option, while studying the impact of the overdispersion parameter *ϕ* by comparing the results for two different values of this parameter. After running different estimators (median on sliding windows of maximum, quasi-maximum likelihood and moment estimators) on several real RNA-Seq data (whole chromosome and genes of various sizes), we fixed *ϕ*_1_=0.3 as a typical value for highly dispersed data as observed in real RNA-Seq data and chose *ϕ*_2_=2.3 for comparison with a reasonably dispersed dataset. For each value, we simulated datasets of size *n* with various densities of number of segments *K*, and only two possible values for the parameter *p*_*J*_: 0.8 on even segments (corresponding to low signal) and 0.2 on odd segments for a higher signal. We had *n* vary on a logarithmic scale between 10^3^ and 10^6^ and *K* between $\sqrt{n}/6$ and $\sqrt{n}/3$. For each configuration, we segmented the signal up to $K_{\max}=\sqrt{n}$ twice: once with the known value of *ϕ* and once with our estimator $\hat{\phi }$ as described above. We started with a window width *h*=15. When the estimate was negative, we doubled *h* and repeated the experience until the median was positive.

Each configuration was simulated 100 times.

For our analysis we checked the run-time on a standard laptop, and assessed the quality of the segmentation using the Rand Index . Specifically, let *C*_*t*_ be the true index of the segment to which base *t* belongs and let ${Ĉ}_{t}$ be the index estimated by the method, then

$$I=\frac{2\sum_{s=1}^{n}\sum_{t>s}\left[1_{C_{t}=C_{s}}1_{{Ĉ}_{t}={Ĉ}_{s}}+1_{C_{t}\neq C_{s}}1_{{Ĉ}_{t}\neq {Ĉ}_{s}}\right]}{\left(n-1)(n-2\right)}.$$

**Figure 3:**
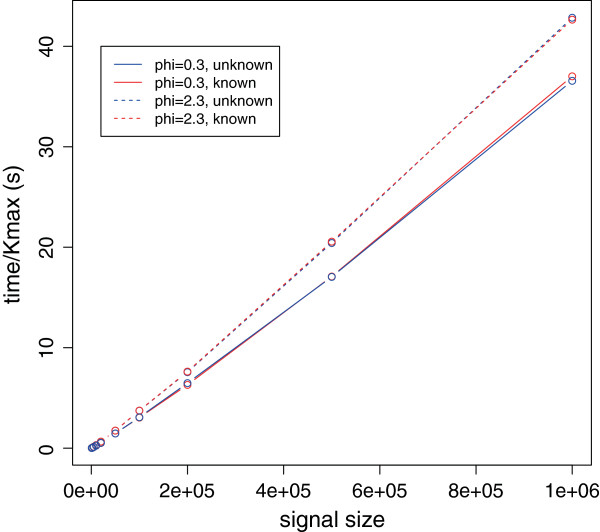
**Run-time analysis for segmentation with negative binomial distribution.** This figure displays the normalized (by *K*_max_) run-time in seconds of the **Segmentor3IsBack** package for the segmentation of signals with increasing length *n*, for two values of the dispersion *ϕ*, and with separate analyses for a known value or an estimated value. While the algorithm is faster for more over-dispersed data, the estimation of the parameter does not slow the processing.

**Figure 4:**
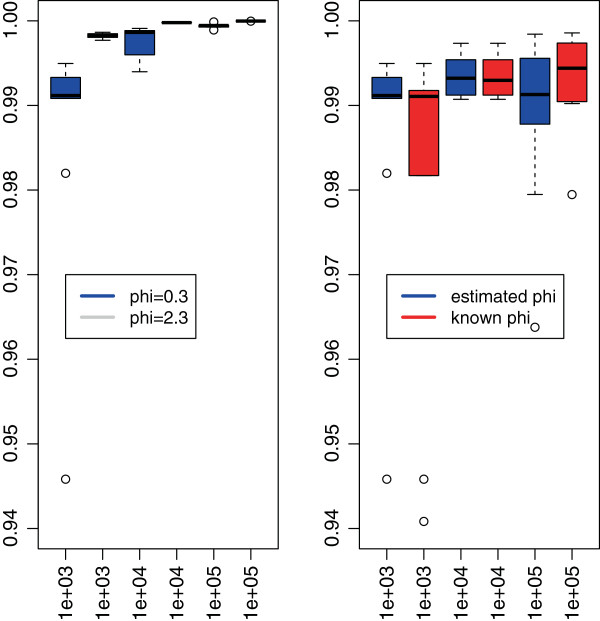
**Rand Index for the quality of the segmentation.** This figure displays the boxplot of the Rand Index computed for each of the hundred simulations performed in the following situations: comparing the values with *ϕ*_1_ and *ϕ*_2_ when estimated (left figure), and comparing the impact of estimating *ϕ*_1_ (right figure). While the estimation does not decrease the quality of the segmentation, the value of the dispersion affects the recovery of the true change-points.

Figure 3 shows, for the particular case of $K=\sqrt{n}/3$, the almost linear complexity of the algorithm in the size *n* of the signal. As the maximal number of segments *K*_max_ considered increased with *n*, we normalized the run-time to allow comparison. This underlines an empirical complexity smaller than $O(K_{\max}n\log n)$, and independent of the value of *ϕ* or its knowledge. Moreover, the algorithm, and therefore the pruning, is faster when the overdispersion is high, a phenomenon already encountered with the *L*^2^ loss when the distribution of errors is Cauchy. However, the knowledge of the true value of *ϕ* does not affect the run-time of the algorithm. Figure 4 illustrates through the Rand Index the quality of the proposed segmentation for a few values of *n*. Even though the indexes are slightly lower for *ϕ*_1_ than for *ϕ*_2_ (see left panel), they range between 0.94 and 1 showing a great quality in the results. Moreover, the knowledge of *ϕ* does not increase the quality (see right panel), which validates the use of our estimator. We can therefore conclude that the run-time of our algorithm without compression is roughly 40×*K*_*max*_×*n*/10^6^s.

### Yeast RNAseq experiment

We applied our algorithm to the segmentation of chromosome 1 of the *S. Cerevisiae* (yeast) using RNA-Seq data from the Sherlock Laboratory at Stanford University [20], publicly available from the NCBI’s Sequence Read Archive (SRA, http://www.ncbi.nlm.nih.gov/sra, accession number SRA048710). We selected the number of segments using our oracle penalty described in the previous section. An existing annotation of translated regions (*i.e.* excluding un-translated regions (UTR)) is available on the Saccharomyces Genome Database (SGD) at http://www.yeastgenome.org, which allows us to validate our results.

With a run-time of 27 minutes without compression, and 5.4 minutes with compression (for a signal length of 230218), we selected 125 segments with the negative binomial distribution. Most of those segments (all but 3) can be related to the official annotation, however as expected segments corresponding to transcribed regions (as opposed to intergenic regions) were found to surround known genes from the SGD due to the difference between transcribed and translated regions. Figure 1 illustrates the result.

**Figure 5:**
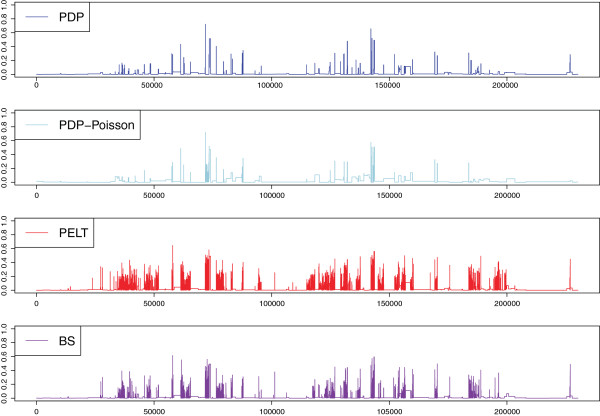
**Comparison of proposed segmentation with annotation and PELT and Binary Segmentation algorithms.** Each figure gives the Hellinger distance between the estimated segmentation of the algorithm (top: PDP with negative binomial, then: PDP with Poisson, third: PELT, and bottom: Binary segmentation) and that of the SGD. The PDP algorithm with the negative binomial distribution seems to outperform other algorithms.

We compared our segmentation with that corresponding to the SGD annotation through the Hellinger distance by fitting a negative binomial distribution on each segment and repeated this comparison with the other two algorithms able to process long count datasets: PELT [16] and Binary Segmentation [6], both implemented in the R package changepoint for the Poisson distribution. For fair comparison, we also used the PDP algorithm for the Poisson loss. Figure 5, together with Table 1 which gives the estimated number of segments, the overall Hellinger score $\left(\underset{t}{\sum }H_{t}/n\right)$ and the number of change-points falling within annotated translated regions, illustrates the result and shows that we outperform the other approaches. Moreover, most of the Hellinger peaks observed can be explained by the fact that we are comparing the annotation of transcribed regions with that of translated regions.

**Table 1:** Comparison of algorithm performance on real data

**Algorithm**	**Number of**	**Hellinger**	**False**
	**segments**	**score**	**positives**
PDPA- negative binomial	125	0.0120	39
PDPA- Poisson	106	0.0187	77
PELT	3416	0.0188	3003
Binary Segmentation	2408	0.0151	2072

Overall Hellinger score of each of the segmentation algorithms, and number of estimated change-points falling within regions annotated as translated (thus considered as false positives).

### Analysis of complex organisms

The issues raised in the analysis of RNA-Seq and DNA-Seq data differ. In the first case, the number of segments that we hope to select is roughly twice the number of expressed exons, therefore the order of *K*_*max*_ varies from 10^2^ (small chromosomes from lower organisms, e.g. yeast) to 10^4^ (large chromosomes from higher organisms, e.g. human). However, when aligned to a reference genome, RNA-Seq data is expected to present large plateaux of zeros at non-coding regions (for instance, 98% of the human genome) and at non expressed regions. The compression option of our algorithm then allows us to reduce the size of the profile by a factor of 10 to 10^3^. Moreover, it is well-known that centromere regions are large non-coding regions where no change-point is expected, and we therefore propose to divide the profile into two parts at such regions. As a proof of concept we ran our algorithm with compression on an RNA-seq profile of the small arm of the 4th chromosome of *Arabidopsis Thaliana* (*n*=4.10^6^, *K*_*max*_=6.10^3^) and selected 4289 segments after a compression factor of 10 and a run-time of 19 hours on a 2.4Ghz computer. The data was kindly provided by some of our collaborators.

DNA-Seq data on the other hand will present much smaller plateaux. While this implies that the compression will be less efficient, the profile can still be summarized into a dataset the length of which will be smaller than the total amount of mapped reads.Most importantly, in these experiments the expected number of segments is drastically smaller as the number of chromosomic aberrations is generally limited to less than one hundred per chromosome, even in pathologies such as cancer.

## Conclusion

Segmentation has been a useful tool for the analysis of biological datasets for a few decades. We propose to extend its application with the use of the Pruned Dynamic Programming algorithm for count datasets such as outputs of sequencing experiments. We show that the negative binomial distribution can be used to model such datasets on the condition that the overdispersion parameter is known and have proposed an estimator of this parameter that performs well in our segmentation framework.

We propose to choose the number of segments using our oracle penalty criterion, which makes the package fully operational. This package also allows the use of other criteria such as AIC or BIC. Similarly, the algorithm is not restricted to the negative binomial distribution but also allows the use of Poisson and Gaussian losses for instance and could easily be adapted to other convex one-parameter losses.

With its empirical complexity of $O(K_{\max}n\log n)$, it can be applied to large signals such as read-alignment of whole chromosomes, and we illustrated its result on a real dataset from the yeast genomes. Moreover, this algorithm can be used as a base for further analysis. For example, [28] use it to initialize their Hidden Markov Model to compute change-point location probabilities.

## Availability and requirements

• Project name: Segmentor3IsBack

• Project home page: http://cran.r-project.org/web/packages/Segmentor3IsBack/index.html

• Operating systems: Platform independent

• Programming language: C++ code embedded in *R* package

• License: GNU GPL

• Any restrictions to use by non-academics: none

## Appendix

**Algorithm 1 The PDP algorithm:**
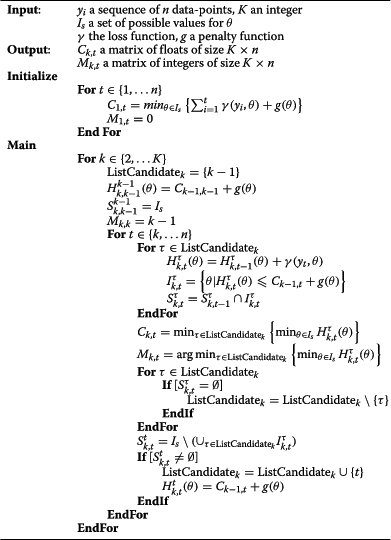


### Proof of Proposition 0.3

#### 

##### 

**Searching for one change-point** Let us first consider a segmentation in 2 segments with a breakpoint at *t*. We define *P*_*t*_(*θ*_1_,*θ*_2_), the cost of this segmentation given some parameter *θ*_1_ for the first segment and *θ*_2_ for the second segment:

$$P_{t}\left(\theta_{1},,,\theta_{2}\right)=\overset{t}{\underset{i=1}{\sum }}\gamma \;\left(y_{i},,,\theta_{1}\right)\;+\;\overset{n}{\underset{i=t+1}{\sum }}\gamma \;\left(y_{i},,,\theta_{2}\right).$$

The optimal cost *P*_*t*_ is:

$$P_{t}={\text{min}}_{\theta_{1}}\left\{\overset{t}{\underset{i=1}{\sum }}\gamma \;\left(y_{i},,,\theta_{1}\right)\right\}\;+\;{\text{min}}_{\theta_{2}}\left\{\overset{n}{\underset{i=t+1}{\sum }}\gamma \;\left(y_{i},,,\theta_{2}\right)\right\}.$$

Having these notations, let us prove the following lemma:

###### **Lemma 0.4.**

• If *t*_1_=1 and *t*_2_=*n* then ∀ *tP*_*t*_≥*C*_1,*n*_

• If *t*_1_=1 and *t*_2_<*n* then ∀ *t*_1_-1≤*t*≤*t*_2_ we have $P_{t}\geq P_{t_{2}}$

• If *t*_1_>1 and *t*_2_=*n* then ∀ *t*_1_-1≤*t*≤*t*_2_ we have $P_{t}\geq P_{t_{1}-1}$

• If *t*_1_>1 and *t*_2_<*n* then ∀ *t*_1_-1≤*t*≤*t*_2_ we have $P_{t}\geq \text{min}\left\{P_{t_{1}-1},P_{t_{2}}\right\}$

Proof

#### 

##### First scenario [ *t*_1_=1 and *t*_2_=*n*]

We have:

$$P_{t}=\mathrm{t.}{\text{min}}_{\theta_{1}}\left\{\gamma (y_{1},\theta_{1})\right\}+(n-t).{\text{min}}_{\theta_{2}}\left\{\gamma (y_{1},\theta_{2})\right\}=C_{1,n}.$$

Thus we get: *P*_*t*_≥*C*_1,*n*_.

#### 

##### Second scenario [ *t*_1_=1 and *t*_2_<*n*]

For any *t* such that *t*≤*t*_2_ we have:

$$\begin{array}{l} P_{t}=\;\mathrm{t.}{\text{min}}_{\theta }\left\{\gamma (y_{1},\theta )\right\}\\ \;+{\text{min}}_{\theta }\left\{\left(t_{2},-,t\right)\gamma \left(y_{1},,,\theta \right)+\overset{n}{\underset{i=t_{2}+1}{\sum }}\gamma \left(y_{i},,,\theta \right)\right\}. \end{array}$$

Thus we have:

$$\begin{array}{l} P_{t}\;\geq \;\mathrm{t.}{\text{min}}_{\theta }\left\{\gamma \left(y_{1},,,\theta_{1}\right)\right\}+\left(t_{2},-,t\right).{\text{min}}_{\theta }\left\{\gamma \left(y_{1},,,\theta \right)\right\}\\ \;+{\text{min}}_{\theta }\left\{\overset{n}{\underset{i=t_{2}+1}{\sum }}\gamma \left(y_{i},,,\theta \right)\right\}. \end{array}$$

And we get $\forall \;t\leq t_{2}\;P_{t}\geq P_{t_{2}}$.

#### 

##### Third scenario [ *t*_1_>1 and *t*_2_=*n*]

We get $\forall \;t_{1}-1\leq t\;P_{t}\geq P_{t_{1}-1}$ by reversing the index and using scenario 2.

#### 

##### Fourth scenario [ *t*_1_>1 and *t*_2_<*n*]

For any *t* such that *t*_1_-1≤*t*≤*t*_2_ we obtain:

$$\begin{array}{l} P_{t}\left(\theta_{1},,,\theta_{2}\right)=\overset{t_{1}-1}{\underset{i=1}{\sum }}\gamma \left(y_{i},,,\theta_{1}\right)\;+\overset{n}{\underset{i=t_{2}+1}{\sum }}\gamma \left(y_{i},,,\theta_{2}\right)\\ \;+\;\left(t,-,t_{1},+,1\right)\gamma \left(y_{t_{1}},,,\theta_{1}\right)+\;\left(t_{2},-,t\right)\gamma \left(y_{t_{1}},,,\theta_{2}\right). \end{array}$$

Thus, for fixed *θ*_1_ and *θ*_2_ and for *t* ∈ [ *t*_1_-1,*t*_2_], *P*_*t*_(*θ*_1_,*θ*_2_) is a linear function of *t*. Thus we obtain that for any *θ*_1_ and *θ*_2_:

$$P_{t}(\theta_{1},\theta_{2})\geq \text{min}\left\{P_{t_{1}-1}(\theta_{1},\theta_{2}),P_{t_{2}}(\theta_{1},\theta_{2})\right\}\geq \text{min}\left\{P_{t_{1}-1},P_{t_{2}}\right\}.$$

As this is true for any *θ*_1_ and *θ*_2_ we get $P_{t}\geq \text{min}\left\{P_{t_{1}-1},P_{t_{2}}\right\}$ ■

#### 

##### Proof of the main proposition

Assume that we have a segmentation *m* in ${ℳ}_{K,n}$ with a breakpoint *τ*_*k*_ in a plateau. Then applying lemma 0.4 on the sequence ${\left\{y_{i}\right\}}_{i\in \left\{\tau_{k-1},\ldots \tau_{k+1}\right\}}$ we see that *τ*_*k*_ can either be discarded or moved to *t*_1_-1 or *t*_2_ without increasing the cost. Thus there exists a segmentation in *K* or fewer segments without any change-point in the plateau such that its optimal cost is *C*_*K*,*n*_. ■

This theorem is more subtle than we might have thought based on our intuition. It does not mean that a change-point in a plateau is never optimal but only that it is not necessary to have change-points in plateaux to achieve optimality.

## Abbreviations

PELT: Pruned exact linear time; PDP: Pruned dynamic programming; AIC: Akaike information criterion; BIC: Bayesian information criterion; NCBI: National Center for Biotechnology Information; SGD: Saccharomyces genome database.

## Competing interests

The authors have no competing interest to declare.

## Authors’ contributions

AC co-wrote the C++ code, wrote the R-package, performed data analysis and co-wrote the manuscript. MK co-wrote the C++ code. EL co-supervised the work and co-wrote the manuscript. GR co-wrote the C++ code, and co-wrote the manuscript. SR co-wrote the manuscript and co-supervised the work. All authors read and approved the final manuscript.
